# GM2 Gangliosidosis AB Variant: A Hidden Truth

**DOI:** 10.7759/cureus.92445

**Published:** 2025-09-16

**Authors:** Inês Noites, Ana Sofia Coelho, Catarina Magalhães, Sandra Ramos, Francisco Laranjeira, Lúcia Lacerda, Ricardo Taipa, Cristina Garrido, Teresa Temudo, Sónia Figueiroa

**Affiliations:** 1 Department of Pediatrics, Hospital do Divino Espírito Santo de Ponta Delgada, Ponta Delgada, PRT; 2 Department of Neuroradiology, Hospital Central do Funchal, Dr. Nélio Mendonça, Serviço de Saúde da Região Autónoma da Madeira (SESARAM), Funchal, PRT; 3 Department of Neuroradiology, Unidade Local de Saúde de Santo António, Porto, PRT; 4 Pediatric Neurology, Department of Pediatrics, Unidade Local de Saúde do Alto Ave, Hospital da Senhora da Oliveira, Guimarães, PRT; 5 Department of Pediatrics, Unidade Local de Saúde da Póvoa de Varzim/Vila do Conde, Póvoa de Varzim, PRT; 6 Biochemical Genetics Unit, Centro de Genética Jacinto Magalhães, Unidade Local de Saúde de Santo António, Porto, PRT; 7 Department of Clinical and Experimental Human Genomics, Unit for Multidisciplinary Research in Biomedicine (UMIB), School of Medicine and Biomedical Sciences (ICBAS) University of Porto, Porto, PRT; 8 Department of Clinical and Experimental Human Genomics and Department of Microscopy, Unit for Multidisciplinary Research in Biomedicine (UMIB), School of Medicine and Biomedical Sciences (ICBAS) University of Porto, Porto, PRT; 9 Department of Neuropathology, Unidade Local de Saúde de Santo António, Porto, PRT; 10 Unit for Multidisciplinary Research in Biomedicine (UMIB), School of Medicine and Biomedical Sciences (ICBAS) University of Porto, Porto, PRT; 11 Department of Neuropediatrics, Centro Materno-Infantil do Norte, Unidade Local de Saúde de Santo António, Porto, PRT

**Keywords:** gm2 activator protein deficiency, gm2a gene, gm2 gangliosidosis ab variant, lysosomal storage disease, neurodegeneration

## Abstract

GM2 gangliosidosis AB variant (GM2AB) is a rare neurodegenerative lysosomal storage disorder with clinical features resembling Tay-Sachs disease but characterized by normal lysosomal β-hexosaminidase A enzyme activity. To date, only 14 cases of the acute infantile form have been reported. To the best of our knowledge, this is the first case of GM2AB in a Portuguese patient reported in the literature. We describe the case of a girl with GM2AB, whose clinical presentation and pathological findings were critical for diagnosis. Post-mortem genetic sequencing identified a pathogenic mutation in homozygosity in the *GM2A* gene, confirming the diagnosis. This case highlights the importance of considering GM2AB in patients with severe neurodegenerative phenotypes and typical pathological findings, even when enzymatic studies are normal. Preserving genetic material post-mortem may allow for diagnosis even years after death, providing critical insights into rare disorders.

## Introduction

GM2 gangliosidoses are a group of rare autosomal recessive lysosomal storage disorders characterized by the pathological accumulation of GM2 gangliosides in the central nervous system (CNS) [1]. These intra-lysosomal deposits lead to progressive cell death, demyelination, and neurodegeneration [2].

The GM2 gangliosidosis AB variant (GM2AB) is distinguished among these disorders by its unique genetic and biochemical characteristics. While Tay-Sachs and Sandhoff diseases are caused by mutations in the *HEXA* and *HEXB* genes, respectively, GM2AB results from mutations in the *GM2A* gene [1,3]. This gene encodes the GM2 activator protein, a non-catalytic cofactor required for the hydrolysis of GM2 gangliosides by the β-hexosaminidase A enzyme complex [2].

A characteristic feature of GM2AB, shared with other GM2 gangliosidoses, is the presence of a cherry-red spot in the retina [1,2,4-9]. This distinctive finding results from ganglioside accumulation in the retinal ganglion cells [1,9], causing the retina to appear whitish and degenerated. In contrast, the macula, devoid of ganglion cells, appears reddish against the pale surrounding retina. This particular feature serves as a crucial diagnostic marker in the clinical evaluation of suspected GM2 gangliosidosis variants [3,4], suggesting that an ophthalmological examination could be useful in the diagnostic workup.

Clinically, GM2AB manifests with a spectrum of symptoms consistent with those observed in Tay-Sachs and Sandhoff diseases [2-4,8]. The acute infantile form of this condition typically presents between four and 12 months of age [5]. Early signs include developmental delay [2-5,8], muscle weakness [1,4,5,8], and exaggerated startle response to auditory stimuli [2,4-6,8]. As the disease progresses, severe neurological deterioration occurs, manifesting as seizures, vision and hearing loss, intellectual disability, and motor dysfunction [4,5,8]. Affected individuals often succumb to the disease within the first few years of life, most commonly from respiratory complications such as recurrent infections and aspiration, secondary to progressive neurological deterioration [5]. The rate of progression and phenotypic severity may vary depending on the predicted impact of the specific mutation on the function of the GM2 activator protein [6,7]. Currently, there is no cure for GM2AB, and treatment focuses on supportive measures to manage symptoms [5].

Pathologically, GM2AB is characterized by significant neurodegeneration within the CNS because of GM2 ganglioside accumulation [1,2,5]. Histological examination reveals widespread neuronal storage, with neurons appearing distended and filled with membranous cytoplasmic bodies [3]. These structures are most prominent in the cerebral cortex, cerebellum, brainstem, and spinal cord. Electron microscopy often reveals multilamellar membrane structures within lysosomes, known as zebra bodies, indicative of lysosomal dysfunction and ganglioside storage [9].

Although the CNS is primarily affected, other tissues, including skin, may also exhibit evidence of ganglioside storage, reflecting the disorder’s multi-systemic nature [5,9]. In GM2AB, routine biochemical assays, including β-hexosaminidase A and B activity, are typically normal, in contrast to Tay-Sachs and Sandhoff disease [4,5]. There are no routine biochemical markers specific for GM2AB. Therefore, diagnosis relies primarily on genetic testing of the *GM2A* gene, which may include single-gene analysis, multigene panels, or comprehensive approaches such as exome sequencing, depending on the clinical context. Specialized functional assays for GM2 activator protein deficiency are available in research settings [2,5,7].

Currently, only 14 cases of the acute infantile presentation of GM2AB have been reported in the literature, highlighting the rarity of this disorder [1,5].

## Case presentation

We present the case of a girl born to healthy, non-consanguineous Portuguese parents. She was delivered via cesarean section at term, with Apgar scores of 6 at one minute and 10 at five minutes. The infant’s early neonatal course was unremarkable, with a normal physical examination and uneventful discharge.

During the first year of life, the patient achieved developmental milestones as expected for age: rolling by four months, sitting unsupported by six months, developing a pincer grasp by nine months, and first words by 11 months. The head circumference was around the 50th percentile at birth, then decreased to below the 10th percentile by five months, preceding subsequent developmental regression. Between 12 and 17 months of age, she experienced a progressive loss of psychomotor skills, initially presenting with cerebellar ataxia and an inability to walk independently. Over time, she lost the ability to sit without support and exhibited choreoathetotic movements of her upper limbs. Regression also affected speech, social interaction, and feeding abilities. By 14 months, she had lost visual attentiveness and began to suffer from generalized tonic-clonic and myoclonic seizures.

Physical examination revealed several notable findings, including microcephaly, non-dysmorphic facial features, episodic deviations of the upper gaze, and a pronounced acoustic startle reflex. Additionally, the child exhibited profound axial hypotonia, pyramidal signs, and a bilateral cherry-red spot on ophthalmologic examination.

Brain MRI findings in the GM2AB variant are highly variable and do not follow a consistent or specific neuroimaging pattern. While some affected infants have been reported with normal brain MRI [1,5,8], others demonstrate abnormal T2 signal intensities in the periventricular white matter and basal ganglia [2,8]. Delayed myelination and T2 hyperintensities in subcortical white matter, basal ganglia, and thalami have also been described [5]. In our patient, the presence of periventricular white matter hyperintensities is in line with previously reported abnormalities, although the overall spectrum of MRI changes in GM2AB remains heterogeneous and nonspecific. A comprehensive laboratory workup (Table 1) showed normal liver and kidney function tests and normal lactate, pyruvate, and ammonia levels. Plasma and urine amino acid chromatography, organic acid excretion, and acylcarnitine profile analysis were all within normal limits. Urinary sulfatides and glycosaminoglycans were also in the normal range. Enzymatic studies conducted on fibroblasts, including assays for β-galactosidase, β-hexosaminidase A and total β-hexosaminidase, sphingomyelinase, galactocerebrosidase, α-neuraminidase, palmitoyl protein thioesterase 1, and tripeptidyl peptidase I, showed normal activity.

**Table 1:** Main laboratory metabolic test results * All values reported as “within normal limits” were assessed by the performing laboratory using standard pediatric reference ranges; exact intervals were not provided for these analyses when results were normal.

Analytical parameters	Value	Reference interval
Lactate	1.8	0.50-2.20 mmol/L
Pyruvate	85.7	34-103 μmol/L
Ammonia	37.5	26-47 μmol/L
Acylcarnitine profile	Within normal limits*	-
Plasma amino acid chromatography	Within normal limits*	-
Urine amino acid chromatography	Within normal limits*	-
β-Galactosidase	322	73.0-585 nmol/h/mg protein
β-Hexosaminidase A	250	185-962 nmol/h/mg protein
Total β-hexosaminidase	4798	3593-30182 nmol/h/mg protein
Galactocerebrosidase	2.6	0.71-3.59 nmol/h/mg protein

**Figure 1:**
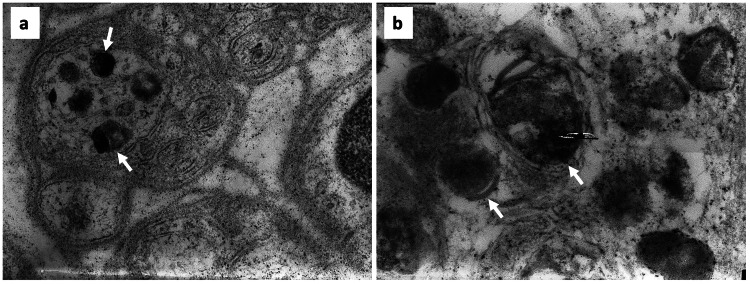
Electron microscopy of skin biopsy showing electron-dense inclusions (arrow) in the cytoplasm of non-myelinated axons. These inclusions are amorphous but occasionally exhibit a lamellar structure at the periphery (a. 20,000x magnification; b. 40,000x magnification).

Skin biopsy analysis, obtained using the routine technique, provided further diagnostic insights. Electron microscopy revealed intraneuronal lamellar inclusions (Figure 1), and toluidine blue staining confirmed the presence of neuronal storage material, consistent with a lysosomal storage disorder. Despite normal enzymatic assay results, these ultrastructural features substantially reinforced the clinical suspicion.

By 35 months of age, her condition had continued to deteriorate. She became unresponsive to environmental stimuli, lacked visual contact and language skills, exhibited flaccid tetraparesis, and responded only to painful stimuli, ultimately succumbing to pneumonia. Despite extensive diagnostic efforts, including comprehensive laboratory and enzymatic studies, a definitive etiology remained unidentified during her lifetime.

Years later, with the advent of *GM2A* gene sequencing at our institution, a homozygous c.333delC p.(Cys112Valfs*7) mutation was identified, confirming the diagnosis of GM2AB. This frameshift variant causes the change of 6 amino acids after position 111 before reaching a premature stop codon. As a result, a truncated protein is produced, approximately in the middle of its natural length, which loses all bisulfide bonds and affects its three-dimensional structure. It is also worth mentioning the presence of several heterozygous polymorphic variants spread across the *GM2A* gene, which corroborate the non-consanguinity of the parents.

At that time, the diagnosis was also confirmed through next-generation sequencing, as the case was included in a research project [10] that aimed to evaluate the use of this methodology for genetic diagnosis.

## Discussion

GM2 gangliosidoses are a group of rare and complex lysosomal storage disorders characterized by intricate clinical, biochemical, and molecular features [1,3,7]. These conditions result from mutations in genes that regulate the breakdown of GM2 ganglioside [2,3], a critical lipid component of neuronal membranes. The degradation of GM2 involves several enzymatic steps [3], with the disruption of this process leading to the accumulation of GM2 gangliosides [2], primarily in the CNS, which causes severe neurodegeneration [1,2,5].

The pathophysiology of GM2 gangliosidosis is closely linked to ganglioside metabolism in neuronal cells, resulting in the CNS being the main site of disease. Therefore, there is a characteristic clinical presentation across the different forms of GM2 gangliosidosis, marked by progressive neurological deterioration [1,4]. Key diagnostic features, such as the presence of a cherry-red spot on retinal examination and early-onset neurodegenerative symptoms, strongly suggest a lysosomal storage disorder [1,4-6,8].

This is the first documented case of this rare variant in a Portuguese patient reported in the literature. In this patient, the normal enzymatic activity of β-hexosaminidases A and B initially masked the diagnosis. At that time, genetic testing for the *GM2A* gene was not available, so a biopsy was performed, which provided supportive pathological findings. The patient’s clinical presentation and pathological findings provided essential clues, prompting further investigation. Currently, when enzyme results are normal despite high clinical suspicion, ophthalmologic evaluation and confirmatory genetic testing should be prioritized.

Ultimately, genetic sequencing conducted years after the patient’s death identified a homozygous c.333delC p.(Cys112Valfs*7) mutation in the *GM2A* gene, confirming the diagnosis of GM2AB. To date, most *GM2A* mutations associated with GM2AB gangliosidosis are missense variants, which are thought to disrupt the GM2 activator protein by impairing its stability or lipid-binding capacity [2,8,9]. The homozygous frameshift mutation identified in this patient leads to a premature stop codon and truncation of the protein, severely compromising its three-dimensional structure. Truncating variants, which include both frameshift and rare nonsense mutations, are generally predicted to result in a more severe loss of function than missense substitutions. Given the small number of reported cases, no clear genotype-phenotype correlation has yet been established [5].

Genetic confirmation clarified the patient’s clinical course and emphasized the pivotal role of molecular diagnostics in identifying complex neurodegenerative disorders and guiding management strategies for affected families.

The diagnostic challenges faced in this case highlight the importance of considering GM2 gangliosidosis in the differential diagnosis, even when conventional enzymatic tests return normal results. The diagnosis of GM2AB, established through gene sequencing, underscores the need for molecular testing in atypical or unresolved cases.

## Conclusions

Currently, GM2 gangliosidosis remains an incurable neurodegenerative disorder with high morbidity and mortality. Nevertheless, an accurate diagnosis is essential for providing optimal supportive care and genetic counselling to affected families. While early recognition of the disease can facilitate appropriate management strategies, the lack of effective therapies emphasizes the need for continued research into potential treatments. This case highlights the critical importance of retaining genetic material from patients with atypical or unresolved cases, particularly those with severe phenotypes. As demonstrated here, advancements in genetic technology can enable definitive diagnoses, even years after a patient’s death. Such diagnoses provide closure for families, inform genetic counseling, and guide decisions regarding future pregnancies, underscoring both the medical and psychosocial value of molecular testing.

## References

[1] Chen Q, Lu F (2023). Multimodal optical imaging and genetic features of AB variant GM2 gangliosidosis: a case report. Front Pediatr.

[2] Sheth J, Datar C, Mistri M, Bhavsar R, Sheth F, Shah K (2016). GM2 gangliosidosis AB variant: novel mutation from India - a case report with a review. BMC Pediatr.

[3] Hall PL, Laine R, Alexander JJ, Ankala A, Teot LA, Lidov HG, Anselm I (2018). GM2 activator deficiency caused by a homozygous exon 2 deletion in GM2A. JIMD Rep.

[4] İnci A, Cengiz Ergin FB, Biberoğlu G, Okur İ, Ezgü FS, Tümer L (2021). Two patients from Turkey with a novel variant in the GM2A gene and review of the literature. J Pediatr Endocrinol Metab.

[5] Xiao C, Toro C, Tifft C (2022). GM2 activator deficiency. GeneReviews® [Internet].

[6] Salih MA, Seidahmed MZ, El Khashab HY (2015). Mutation in GM2A leads to a progressive chorea-dementia syndrome. Tremor Other Hyperkinet Mov (N Y).

[7] Martins C, Brunel-Guitton C, Lortie A, Gauvin F, Morales CR, Mitchell GA, Pshezhetsky AV (2017). Atypical juvenile presentation of GM2 gangliosidosis AB in a patient compound-heterozygote for c.259G > T and c.164C > T mutations in the GM2A gene. Mol Genet Metab Rep.

[8] Renaud D, Brodsky M (2016). GM2-gangliosidosis, AB variant: clinical, ophthalmological, MRI, and molecular findings. JIMD Rep.

[9] Toro C, Zainab M, Tifft CJ (2021). The GM2 gangliosidoses: unlocking the mysteries of pathogenesis and treatment. Neurosci Lett.

[10] Fernández-Marmiesse A, Morey M, Pineda M (2014). Assessment of a targeted resequencing assay as a support tool in the diagnosis of lysosomal storage disorders. Orphanet J Rare Dis.

